# Note on a differentiation formula, with application to the two-dimensional Schrödinger equation

**DOI:** 10.1371/journal.pone.0171444

**Published:** 2017-02-08

**Authors:** Alexander Pikovski

**Affiliations:** JILA, NIST & Department of Physics, University of Colorado, Boulder, CO, United States of America; University of Calgary, CANADA

## Abstract

A method for obtaining discretization formulas for the derivatives of a function is presented, which relies on a generalization of divided differences. These modified divided differences essentially correspond to a change of the dependent variable. This method is applied to the numerical solution of the eigenvalue problem for the two-dimensional Schrödinger equation, where standard methods converge very slowly while the approach proposed here gives accurate results. The presented approach has the merit of being conceptually simple and might prove useful in other instances.

## Introduction

A discrete representation of the derivative of a function is often required in numerical computations. In order to solve an equation involving derivatives, one introduces a grid (mesh) and approximates the original equation by a system of equations which use the values of the unknown function on the grid points. Such a method can be used to solve, for example, a two-point boundary-value problem or an eigenvalue problem involving an ordinary differential equation.

Numerical methods which discretize derivatives on a grid rely on the fact that the discretization error decreases as the grid spacing is decreased. However, the accuracy of discretization can vary considerably. If the unknown function changes rapidly in a short interval, usually many grid points are required in order to capture its behavior sufficiently well in this region. In some cases, it is not practical to decrease the grid spacing until convergence is reached because the rate of convergence is too slow. Instead, one has either to re-formulate the problem to make it more well-behaved or to adapt the numerical method to the particular problem. In adapting the numerical method to the problem, one may proceed along two directions. One may construct a grid which better approximates the unknown function [1, 2], e.g. which is denser in the region where the function changes rapidly. Another way is to keep a generic grid, but to adapt the expressions which are used to discretize the derivatives in the particular problem; this has been often discussed in the context of boudary layers [1, 3].

In this paper, we present a conceptually simple method to discretize derivatives of a function which is close to another function. The presented results can be viewed as a generalization of a formula of Zadorin [3].

The method presented here uses modified divided differences. The subject of divided differences is classical and well-known. Here, after a short presentation of divided differences, we discuss modified divided differences and give the differentiation formulas. These are applied to the numerical solution for the two-dimensional Schrödinger equation and compared to other techniques, showing that the present method is superior.

## Divided differences and differentiation formulas

The divided differences (of order 1, 2, …) of a function *f* are defined by [4, 5]:

$$f\left(x_{0},x_{1}\right)=\frac{f\left(x_{1}\right)-f\left(x_{0}\right)}{x_{1}-x_{0}},$$ (1)

$$f\left(x_{0},x_{1},x_{2}\right)=\frac{f\left(x_{1},x_{2}\right)-f\left(x_{0},x_{1}\right)}{x_{2}-x_{0}},\;\ldots$$ (2)

They can be shown to be symmetric in their arguments. Another property is that the *k*-th order divided differences of a polynomial of degree *n* result in a polynomial of degree *n* − *k* (and zero if *k* > *n*). This is seen [4, 5] by considering the first divided difference of *x*^*n*^ at points *x* and *a*, which is

$$\frac{x^{n}-a^{n}}{x-a}=x^{n-1}+x^{n-2}a+\ldots +a^{n-1}.$$ (3)

For any function *f* there is the exact identity known as Newton’s formula:

$$\;\;f\left(x\right)\;=\;f\left(a_{0}\right)+\left(x-a_{0}\right)f\left(a_{0},a_{1}\right)+\left(x-a_{0}\right)\left(x-a_{1}\right)f\left(a_{0},a_{1},a_{2}\right)\;+\ldots +\left(x-a_{0}\right)\cdots \left(x-a_{n-1}\right)f\left(a_{0},\ldots ,a_{n}\right)+E\left(x\right)$$ (4)

with

$$E\left(x\right)=\left(x-a_{0}\right)\cdots \left(x-a_{n}\right)f\left(a_{0},\ldots ,a_{n},x\right)$$ (5)

$$=\left(x-a_{0}\right)\cdots \left(x-a_{n}\right)\frac{f^{n+1}\left(\xi \right)}{(n+1)!}$$ (6)

where *ξ* is some number lying between the largest and the smallest of the arguments (*a*_0_, …, *a*_*n*_, *x*). If the error term *E*(*x*) is omitted from Eq (4), one obtains Newton’s interpolating polynomial (of order *n*) for the function *f* at the points *a*_0_, …, *a*_*n*_.

Expressions for the discretized derivative can be deduced from Newton’s interpolation formula by differentiating and, if needed, by further manipulations [4]. Here we only consider the following two formulas. Considering Eq (4) up to the linear term (i.e. with higher terms omitted), differentiating it with respect to *x*, and putting *a*_0_ = *x*, *a*_1_ = *x* + *h* gives

$$f^{\prime }\left(x\right)\approx f\left(x,x+h\right)=\frac{f(x+h)-f(x)}{h}.$$ (7)

The same procedure up to quadratic terms and differentiating twice with *a*_0_ = *x* − *g*, *a*_1_ = *x*, *a*_2_ = *x* + *h* gives

$$f^{\prime \prime }\left(x\right)\approx 2f\left(x-g,x,x+h\right).$$ (8)

It is helpful to know that the second divided differences can also be written in the form

$$f\left(a_{0},a_{1},a_{2}\right)=\frac{f(a_{0})}{\left(a_{0}-a_{1}\right)\left(a_{0}-a_{2}\right)}+\frac{f(a_{1})}{\left(a_{1}-a_{0}\right)\left(a_{1}-a_{2}\right)}+\frac{f(a_{2})}{\left(a_{2}-a_{0}\right)\left(a_{2}-a_{1}\right)}.$$ (9)

For *g* = *h* Eq (8) gives the well-known

$$f^{\prime \prime }\left(x\right)\approx \frac{f(x-h)-2f(x)+f(x+h)}{h^{2}}.$$ (10)

The differentiation formulas of Eqs (7) and (8) are exact for polynomials up to degree one and two, respectively, as follows from the property of divided differences of polynomials mentioned above. The error term of these approximations to the derivatives can be found from Newton’s formula, differentiating the error term of Eqs (5) or (6). It is seen that the error of the approximation Eq (8) is proportional to *f*′(*ξ*) and *f*″(*ξ*) evaluated at some points *ξ* in the interval *x* − *g* < *ξ* < *x* + *h*.

## Modified divided differences and differentiation formulas

Modified divided differences with respect to a function Φ will now be introduced. The function Φ(*x*) is assumed to be strictly increasing. Define the modified divided differences with respect Φ(*x*) by

$$f_{\Phi }\left(x_{0},x_{1}\right)=\frac{f\left(x_{1}\right)-f\left(x_{0}\right)}{\Phi \left(x_{1}\right)-\Phi \left(x_{0}\right)},$$ (11)

$$f_{\Phi }\left(x_{0},x_{1},x_{2}\right)=\frac{f_{\Phi }\left(x_{1},x_{2}\right)-f_{\Phi }\left(x_{0},x_{1}\right)}{\Phi \left(x_{2}\right)-\Phi \left(x_{0}\right)},\;\ldots$$ (12)

Clearly, for Φ(*x*) = *x* they reduce to the ordinary divided differences. We also have

$$f_{\Phi }\left(x_{0},\ldots x_{n}\right)=\left(f\circ \Phi^{-1}\right)\left(\Phi \left(x_{0}\right),\ldots \Phi \left(x_{n}\right)\right),$$ (13)

noting that the inverse function Φ^−1^ exists since Φ(*x*) is strictly increasing. Therefore, the properties of the modified divided differences can be deduced from the properties of the ordinary divided differences. The *k*-th modified divided differences of a polynomial of degree *n* in the variable *y* = Φ(*x*) give a polynomial in *y* of order *n* − *k*, since the equivalent of Eq (3) is $\frac{\Phi {\left(x\right)}^{n}-\Phi {\left(a\right)}^{n}}{\Phi (x)-\Phi (a)}$. Newton’s formula for the modified divided differences reads

$$\;\;f\left(x\right)=f\left(a_{0}\right)+\left(\Phi \left(x\right)-\Phi \left(a_{0}\right)\right)f_{\Phi }\left(a_{0},a_{1}\right)+\left(\Phi \left(x\right)-\Phi \left(a_{0}\right)\right)\left(\Phi \left(x\right)-\Phi \left(a_{1}\right)\right)f_{\Phi }\left(a_{0},a_{1},a_{2}\right)+\ldots$$ (14)

and the higher terms and the error term (omitted here for brevity) are constructed as in Eqs (4) and (5).

The two-point formula for the approximate derivative is obtained by differentiating Eq (14) up to the linear term and putting *a*_0_ = *x*, *a*_1_ = *x* + *h*:

$$f^{\prime }\left(x\right)\approx \Phi^{\prime }\left(x\right)f_{\Phi }\left(x,x+h\right)=\Phi^{\prime }\left(x\right)\frac{f(x+h)-f(x)}{\Phi (x+h)-\Phi (x)}.$$ (15)

This formula [Eq (15)] as well as the linear term of Eq (14) were suggested by Zadorin [3]. Differentiating Eq (14) twice, up to the three-point term, and putting *a*_1_ = *x* gives

$$f^{\prime \prime }\left(x\right)\approx 2\left[\Phi^{\prime }{\left(x\right)}^{2}+\frac{1}{2}\Phi^{\prime \prime }\left(x\right)\left(\Phi \left(x\right)-\Phi \left(a_{0}\right)\right)\right]\;f_{\Phi }\left(a_{0},x,a_{2}\right)+\Phi^{\prime \prime }\left(x\right)f_{\Phi }\left(a_{0},x\right).$$ (16)

The differentiation formulas Eqs (15) and (16) are exact for polynomials up to degree one and two, respectively, in the variable *y* = Φ(*x*).

## Application: Eigenvalue problem for the 2D Schrödinger equation

As an application of the formulas of the previous section, we consider the eigenvalue problem for the two-dimensional Schrödinger equation. Diverse physical systems, such as solid-state hetersotructures [6, 7], layered superconductors [8], as well as the recently discovered two-dimensional crystals [9], are described quantum-mechanically by the Schrödinger equation in two spatial dimensions. Many physical properties of these systems are determined by the single-particle energies, i.e. the quantum-mechanical bound states of a particle in an external potential. A large variety of methods for the numerical solution of eigenvalues of Schrödinger have been discussed in the literature, the reader may consult Ref. [10] for a long list of references.

Bound states are solutions of the time-independent Schrödinger equation, which in two dimensions reads $\left(-\nabla^{2}+V\left(\vec{r}\right)\right)\Psi \left(\vec{r}\right)=E\Psi \left(\vec{r}\right)$, where $\vec{r}=\left(x,y\right).$ The problem is to find the energies (eigenvalues) *E* and the wave functions (eigenvectors) $\Psi (\vec{r})$ in a given external potential *V*. We assume that the potential is radially symmetric $V=V(|\vec{r}|)$. Using polar coordinates (*r*, *φ*) and writing $\Psi \left(\vec{r}\right)=\sum_{\ell =-\infty }^{\infty }e^{i\ell \phi }\psi \left(r\right)/\sqrt{r}$, the problem is reduced to an ordinary differential equation for each *ℓ*, the so-called radial equation. Here we consider only the radial equation for *ℓ* = 0 (this corresponds to the lowest bound state), it reads

$$\left\{-\frac{d^{2}}{dr^{2}}-\frac{1/4}{r^{2}}+V\left(r\right)\right\}\psi \left(r\right)=E\psi \left(r\right).$$ (17)

The boundary conditions for this eigenvalue problem for bound states are

ψ(0)=0,ψ(∞)=0. (18)

The straightforward method to solve this eigenvalue problem numerically is to discretize the Eq (17) on a grid and solve the resulting matrix eigenvalue problem. The discretization of the second derivative can be done using Eq (8). It is found, however, that this method produces very inaccurate results. The convergence of the numerical values of the eigenvalues is very slow as the number of grid points is increased, both for a uniform and a non-uniform grid (see Table 1). The wave function obtained using this method is, in fact, inaccurate for points close to the origin. The reason for this behavior is that the exact solution of Eq (17) behaves for small *r* as

$$\psi \left(r\right)∼\sqrt{r}.$$ (19)

This leads to large errors in the discretization formula of Eq (8), since the error is proportional to the derivatives of the function [cf. Eq (6)] which are here very large for small *r* [cf. Eq (19)].

**Table 1:** Comparison of different numerical methods.

Discretization	Grid	*N*	*h*	*E*_0_	Accuracy
Eq (10)	uniform	1000	0.005	2.256358	1.1282
		10000	0.0005	2.198202	1.0991
		100000	0.00005	2.161484	1.0807
		1000000	0.000005	2.136218	1.0681
Eq (8)	non-uniform	1000		2.120170	1.0601
		10000		2.094154	1.0471
		100000		2.077388	1.0387
		1000000		2.065690	1.0329
Eq (16) for $\Phi \left(x\right)=\sqrt{x}$	uniform	100	0.05	1.996716	0.9984
		500	0.01	1.999840	0.9999

Comparison of numerical methods for the eigenvalue problem of the two-dimensional Schrödinger equation, for the exactly solvable case of the two-dimensional harmonic oscillator. The problem is discretized in the range 0 < *r* < *x*_*m*_ = 5 on a grid with *N* points. For a uniform grid the spacing is *h*, the non-uniform grid points are taken to be *n*^2^*x*_*m*_/*N*^2^, *n* = 0…*N*. The numerically found lowest eigenvalue (ground state) is denoted *E*_0_ and the accuracy is *E*_0_ divided by the exact value *E*_0_ = 2.

The discretization formula derived previously can be used for this problem to obtain accurate results. One may use Eq (16) with $\Phi \left(x\right)=\sqrt{x}$ to obtain a discretization for the second derivative, and use it to solve the eigenvalue problem for the two-dimensional Schrödinger Eq (17).

To assess the accuracy of the numerical methods, we compare the results of the numerical calculation of eigenvalues with an exact solution. For *V*(*r*) = *r*^2^, the eigenvalue problem of Eq (17) can be solved exactly (this is the harmonic oscillator in two dimensions). The lowest eigenvalue is *E*_0_ = 2 (in the units used here) and the corresponding eigenfunction is $\psi \left(r\right)=\sqrt{r}e^{-r^{2}/2}$. Table 1 compares the results for this problem obtained with different discretization schemes. We compare the standard discretization on a uniform grid [Eq (10)] and on a non-uniform grid [Eq (8)] with the results obtained by discretizing using modified divided differences, Eq (16), with $\Phi \left(x\right)=\sqrt{x}$. It is seen that the discretization formula with modified divided differences is clearly superior to the two other methods.

In the case of a one-dimensional and three-dimensional Schrödinger equation, the numerical problem discussed here does not arise. Standard numerical discretization methods, without corrections, can be applied there and produce accurate results.

Finally, it should be noted that the eigenvalue problem for two-dimensional Schrödinger equation can also be treated with other methods. A substitution $\phi \left(r\right)=\psi \left(r\right)/\sqrt{r}$ brings Eq (17) to a form where the wave function is better behaved at the origin, and the boundary condition of Eq (18) has to be modified. Then the standard discretization procedure, even on a uniform grid, produces accurate results. However, often the vanishing boundary conditions of Eq (18) are more convenient for numerical work, and in this case our technique will be useful.

## Conclusions

A method of discretizing derivatives of a function is presented which derives from the notion of modified divided differences with respect to an increasing function Φ(*x*). The resulting formulas for derivatives correspond to polynomial interpolation in the variable *y* = Φ(*x*). The expressions generalize a formula due to Zadorin [3]. The method is found to be useful for the eigenvalue problem for the two-dimensional Schrödinger equation. For an exactly solvable case, the method is compared to the usual discretization formulas and found to be more accurate by orders of magnitude. The technique is simple conceptually and might prove of use in other problems.
